# Depression and Anxiety Among Adolescents in Northern Sudan: A School-Based Cross-Sectional Study

**DOI:** 10.3390/medicina61020228

**Published:** 2025-01-27

**Authors:** Ahmed A. Hassan, Maysoon B. Idrees, Abdullah Al-Nafeesah, Hatim Y. Alharbi, Ashwaq AlEed, Ishag Adam

**Affiliations:** 1 Faculty of Medicine, University of Khartoum, Khartoum P.O. Box 102, Sudan; aa801181@gmail.com (A.A.H.); dr.maisoon_idress@yahoo.com (M.B.I.); 2 Department of Pediatrics, College of Medicine, Qassim University, Buraydah 52571, Saudi Arabia; a.alnafeesah@qu.edu.sa; 3 Department of Psychiatry, College of Medicine, Qassim University, Buraydah 52571, Saudi Arabia; hy.alharbi@qu.edu.sa; 4 Department of Obstetrics and Gynecology, College of Medicine, Qassim University, Buraydah 52571, Saudi Arabia; ia.ahmed@qu.edu.sa

**Keywords:** adolescent, prevalence, associated factors, depression, anxiety, gender

## Abstract

*Background and Objective*: Adolescents’ mental health, including depression and anxiety, represent a global public health problem. However, there is a paucity of data on depression and anxiety among adolescents in Sudan. Therefore, the current study aimed to investigate the prevalence and associated factors for depression and anxiety among adolescents in Northern Sudan. *Methods*: This school-based cross-sectional study was conducted at randomly selected schools from August to September 2022 in Almatamah, River Nile State, Sudan. Sociodemographic and clinical data were collected using a face-to-face questionnaire. Depression and anxiety were measured using the questionnaire tools of the Patient Health Questionnaire (PHQ-9) and the General Anxiety Disorder (GAD-7), respectively. Univariate and multivariate binary analyses were performed to determine the associated factors of depression and anxiety using the adjusted odd ratios (AOR) and 95.0% confidence interval (CI). *Results*: Of 384 adolescents, 178 (46.4%) and 206 (53.6%) were boys and girls, respectively. The median (interquartile range [IQR]) of age was 15.1 (14.0–16.3) years old. Of the total adolescents, 311 (81.0%), 42 (10.9%), 20 (5.2%), 7 (1.8%), and 4 (1.0%) had no, mild, moderate, moderate to severe, and severe depression, respectively. Of the total, 31 (8.1%) adolescents had moderate or severe depression (depression score ≥ 10). In multivariable binary analysis, female gender (AOR = 5.35, 95% CI 1.67–17.09) and anxiety (AOR = 25.98, 95% CI 7.68–87.90) were associated with increased odds of depression; there was no association between age, father’s education, and BMI for age Z-score and depression. Of the 384 adolescents, 320 (83.3%), 47 (12.2%), 11 (2.9%), and 6 (1.6%) had no mild, moderate, and severe anxiety, respectively. Of the total, 17 (4.4%) had moderate and or severe anxiety (anxiety score ≥ 10). In multivariable logistic regressions, while family history of mental disorder (AOR = 6.31, 95% CI 1.36–29.27), being anemic (AOR = 3.95, 95% CI 1.19–13.12), and depression (AOR = 29.03, 95% CI 7.52–112.05) were associated with increased odds of anxiety; there was no association between age, gender, father’s occupation, and BMI for age Z-score and anxiety. *Conclusions*: The findings indicate that 1 in 10 adolescents in Northern Sudan experiences at least one mental disorder, encompassing depression, anxiety, or a combination of both. Notably, female adolescents are at a higher risk for depression, while factors such as a family history of mental disorders and anemia significantly contribute to anxiety levels. To address these pressing mental health concerns, it is imperative to implement inclusive and holistic programs that incorporate nutritional support, integrate mental health education into school curricula, and introduce community-based interventions aiming to enhance mental well-being among all adolescents, irrespective of gender.

## 1. Introduction

The World Health Organization (WHO) defined adolescence as “the phase of life between childhood and adulthood, ages 10 to 19 years” [1]. The adolescent period is a fundamental stage of human development and acts as a foundation for future health [1]. There is an increasing trend of adolescent mental health disorders, including depression and anxiety, at the global level, including in Sub-Saharan Africa [2]. According to the WHO, globally, 1 in 7 adolescents experiences a mental disorder, accounting for 13% of the global burden of disease in this age group [3]. In Sub-Saharan Africa, it is estimated that one-quarter (26.9%) of adolescents have depression, and about one-third (29.8%) of them have anxiety disorders [4]. Adolescents’ mental health disorders, including depression and anxiety, have both short and long-term impacts [5]. Short-term impacts of depression and anxiety among adolescents include poor academic performance and, as a consequence, school dropout [6,7,8], smoking [7,9], and alcohol consumption [7]. Moreover, a study shows that children and adolescents with comorbid depression and anxiety are at the highest risk for having more adverse outcomes, including substance misuse, in young adulthood, i.e., 24 years old [5].

In Khartoum State, Central Sudan, 54% of high school students taking the Sudanese National Board examination had anxiety ranging from high to extreme anxiety, and 25% had severe depression [6]. In Northwest Ethiopia, the prevalence of depression and anxiety among adolescents was 41.4% and 66.7%, respectively [7]. Depression and anxiety are co-occurrences, i.e., comorbidity in children and adolescents [5,9,10]; therefore, they share similar factors. Several factors, such as age [11], being female [7,11,12], urban residency [13], smoking [9], obesity [14,15,16], anemia [16,17], maternal level of education [6], smoking [7], are associated with depression and anxiety among adolescents. To achieve good health, adolescents’ mental health needs must be addressed at all levels and by all involved parties. To improve adolescents’ mental health and well-being, researchers recommend a preventive approach. For example, raising awareness via optimum screening tools, early detection, and treatment of mental health disorders, including depression and anxiety, among children and adolescents could prevent the short- and long-term impacts of these disorders and, as a result, avoidance of their negative impacts on health and economy. This preventive approach is required in low- and middle-income countries (LMICs), especially in Sudan, where resources are limited. Based on the WHO estimation, more than 20% of the population in Sudan are adolescents [18]. Moreover, among population groups, adolescents are at higher risk of mental health disorders, especially those living in poverty [4]. The current war in Sudan also negatively impacts adolescents’ mental health [19].

It is clear that both depression and anxiety among adolescents are global public health problems and necessitate an urgent global call for action. Therefore, as a practical step to address adolescents’ mental health and its predictors on a worldwide scale, a thorough understanding of the local context is needed. To achieve this, first, the prevalence of both depression and anxiety among adolescents in the community and its associated factors must be investigated. Based on community-based data, appropriate healthcare measures can be applied to ensure good adolescent health in the present and adulthood.

There is limited published data on adolescents’ mental health in Sudan [6,8], and no such data have been published in the proposed region (Northern Sudan). In Sudan, there is a shortage of mental health professionals, including sociologists, psychologists, nurses, and primary care doctors, to provide early interventions at the grassroots, especially in rural regions [20]. In addition, conducting such a study will act as a foundation to address the population’s mental health in Sudan, especially among children and adolescents. Therefore, the current study aimed to investigate the prevalence and associated factors of depression and anxiety among adolescents in Northern Sudan.

## 2. Materials and Methods

### 2.1. Study Design and Setting

This school-based cross-sectional study was conducted among 384 adolescents. This study was conducted at governmental schools in the locality of Almatamah in Northern Sudan from August to September 2022. Almatamah is located in the River Nile State, Northern Sudan. Almatamah is approximately 100 km from Khartoum City (the capital of Sudan). The Wad Hamid district, Almatamah locality, was selected for this study for several reasons; among them, it is an understudied area, and our previous study showed some concerns about adolescents’ health (e.g., vitamin D deficiency and poor academic performance concerns) in the Wad Hamid district, Almatamah locality [21]. In the present study, strengthening the reporting of observational studies in epidemiology (STROBE) guidelines were strictly followed [22].

### 2.2. Sampling Technique

Almatamah has three districts, one being the Wad Hamid district. In the Wad Hamid district, there are 16 governmental schools (no private schools) for both boys and girls. Six schools (three for each gender) out of the sixteen schools were randomly selected using simple random selection via the lottery method. A total of 5190 students were registered in the six schools. A total of 384 students (the desired sample size) were taken from the selected schools. The number of students taken from each school depends on the total number of students in the specific school, i.e., probability proportional to size (PPS). Therefore, schools with more students contributed more to the sample. In each school, the assigned sample size was selected using a simple random technique (lottery method) from the list of students in the school.

### 2.3. Inclusion and Exclusion Criteria

The inclusion criteria included healthy (apparently) adolescents aged 10 to 19 years old. Students with younger ages (<10 years old) or older ages (>19 years old), students who did not give their consent for participation in the study, and those who were sick, previously diagnosed with any mental disorders (e.g., bipolar disorder and schizophrenia), receiving any mental health disorder medications, pregnant, or lactating girls were excluded from participating in this study. For example, pregnant and lactating adolescents were excluded from this study because they face unique mental health challenges, necessitating separate research to focus on their specific needs and vulnerabilities.

### 2.4. Sample Size Calculation

An OpenEpi Menu software was used to compute the desired sample size [23]. A sample of 384 adolescents was computed for the present study. There was no previous study in the study region. Therefore, the maximum (50.0%) of the event (depression/anxiety) was assumed to have the optimum sample size using the equation, ‘n = Z^2^pq/d^2^, in which *q* = (1 − *p*), *Z*1 − α = confidence interval (CI) of 95% = 1.96, and *d* = margin of error of 5% = 0.05)’.

### 2.5. Study Variables and Measures

The questionnaire was developed from previous studies, including those of Sudan [6,7,8,9,11,12,13,14,15,24,25,26,27]. The questionnaire included data on sociodemographic characteristics, such as age in years, gender (male or female), parental educational levels (<secondary or ≥secondary), mother’s occupational status (housewife or employed), father’s occupational status (skilled worker or unskilled worker), smoking habit (yes or no), and clinical data such as anemia. The investigators trained three medical research assistants to conduct data collection.

After the participants and their parents/guardians agreed to participate and signed an informed consent form, the medical research assistants approached the selected students. The selected students were informed about the aims and all necessary information, including the voluntary participation in the study and their right to withdraw from the study at any time without giving any reason/s, and the preventive measures taken to ensure the privacy and confidentiality of the participants, such as excluding personal identifiers during data collection. Depression, anxiety, weight, and height were measured using the standard procedures as detailed below. The sociodemographic, clinical, and body mass index (BMI) were considered secondary outcomes. Depression and anxiety were considered as the primary outcomes independently. All measurements were taken under the same conditions for all participants (e.g., at the same time of day) to avoid differences in the data. The study was carried out during the first semester.

### 2.6. Outcomes Measurements

The present study used the Patient Health Questionnaire (PHQ)-9 to measure symptoms of depression. This questionnaire (PHQ-9) is a nine-item depression screening instrument developed by Kroenke et al. [28]. Responses are measured using a four-point Likert-type scale in which responses are summed using a range from (0) for ‘Not at all’ to a score of (3) for ‘Almost every day’. The score ranged from 0 to 27. Therefore, higher PHQ-9 scores indicate higher levels of depression symptoms and vice versa. Furthermore, total scores of depression were categorized into no depression (0–4), mild (5–9), moderate (10–14), moderate to severe (15–19), and severe depression (20–27) [28]. The PHQ-9 has adequate validity for screening depression and adequate internal consistency reliability in a variety of populations, including adolescents [29,30]. For example, Fonseca-Pedrero et al. concluded that the PHQ-9 is a reliable and applicable tool for assessing self-reported depressive symptoms in both clinical and nonclinical settings, including school settings; they recommended PHQ-9 to be used as a screening tool for universal early detection and follow up of depression symptoms during adolescence [30].

On the other hand, the Generalized Anxiety Disorder (GAD-7) questionnaire was used to measure anxiety symptoms in this study. This questionnaire (GAD-7) is a seven-item tool developed by Spitzer et al. [31]. The items of GAD-7 evaluate symptoms of panic disorder and generalized anxiety disorder over the past two weeks. The responses on GAD are measured using a four-point Likert-type scale, in which responses are scored between a score of (0) for ‘Not at all’ and a score of (3) for ‘Almost every day’. The score ranged from 0 to 21. Therefore, the higher the GAD scores indicate the higher levels of anxiety and vice versa. Furthermore, the total scores of anxiety were categorized into no (0–4), mild (5–9), moderate (10–14), and severe anxiety (15–21) [31]. The GAD-7 had good reliability, criterion, construct, and procedural validity. Both PHQ-9 and GAD-7 have been widely used in similar contexts, including African and Arab populations [13,26,27,32,33]. In the present study, the cutoff of PHQ-9 ≥ 10 and GAD-7 ≥ 10 are used for depression and the high level of anxiety among adolescents for the multivariate logistic regression analysis based on the previous studies [13,27]. Other tools are used to measure mental health disorders for depression, such as the Center for Epidemiological Studies Depression Scale for Children [CES-DC] (54), and anxiety, such as Screen for Child Anxiety and Emotion-related Disorder [SCARED]—child version [34]. In this study, the authors utilized the PHQ-9 and GAD-7 questionnaires, which have previously been shown to be culturally and linguistically appropriate for Sudanese adolescents [13,27,35].

### 2.7. Weight and Height Measurements

The students’ weights were measured in kilograms (kg) using the standard procedures, which were well-calibrated scales adjusted to zero before each measurement. Weight was measured to the nearest 100 g. The students stood with minimal movement, with their hands by their sides. In addition, shoes and excess clothing were removed. Height was measured to the nearest 0.1 cm, with the students standing straight with their backs against the wall and feet together. Furthermore, the BMI for the age Z-score was determined based on the WHO’s standards [36].

### 2.8. Blood Samples Processing

For each student, 3 mL of blood was taken for hemoglobin analysis under aseptic conditions. These samples were used as part of a complete blood count. An automated hematology analyzer (Sysmex KX-21, Kobe, Japan) was used to measure hemoglobin levels, as described in our previously published work [37]. Based on the WHO’s recommendation for adolescents, a hemoglobin concentration of a cutoff of <12 g/dL in females and <13 g/dL for males was considered to diagnose anemia [38].

### 2.9. Statistical Analysis

The collected data were entered into IBM Statistical Product and Service Solutions (SPSS) for Windows (version 22.0; SPSS Inc., New York, NY, USA) for analysis. Continuous data such as age, BMI, and hemoglobin were evaluated for normality using the Kolmogorov–Smirnov test, and they were found to be non-normally distributed. Therefore, they are expressed as median (interquartile range [IQR]).

Adjusted multivariate analysis was performed on severe depression and severe anxiety as categorical variables (for multivariate), as dependent variables, independently, sociodemographic (age, gender, parents’ educational occupation status, smoking habit), and anemia as independent variables. Furthermore, variables in the univariate analysis with a *p*-value of <0.2 were entered into the multivariate logistic regression to adjust for covariates. Adjusted odds ratios (AORs), 95% confidence intervals (CIs), coefficients, and standard errors were calculated as they were applied. A two-sided *p*-value of <0.05 was considered statistically significant.

## 3. Results

### 3.1. General Characteristics of the Studied Participants

From the total enrolled adolescents (*n* = 384), 178 (46.4%) and 206 (53.6%) were boys and girls, respectively. The median (IQR) age was 15.1 (14.0–16.3) years old. Median (IQR) of BMI for age Z-score and hemoglobin were −0.65 (−1.63–0.36) and 13.0 (12.3–14.1) g/dL, respectively. Of the total 384 adolescents, 242 (63.0%) mothers had an education level of secondary and above, and 251 (65.4%) fathers’ had an education level of secondary and above. Only about 1 in 10, 34 (8.9%) of the participants’ mothers were employed. Of the total (*n* = 384) participants, 12 (3.1%) were cigarette smokers. About 1 in 10, 34 (8.9%) of the participants had a family history of mental disorder. Almost one-quarter of the participants, 95 (24.7%), were anemic (Table 1).

### 3.2. Prevalence and Associated Factors for Depression

**Figure 1:**
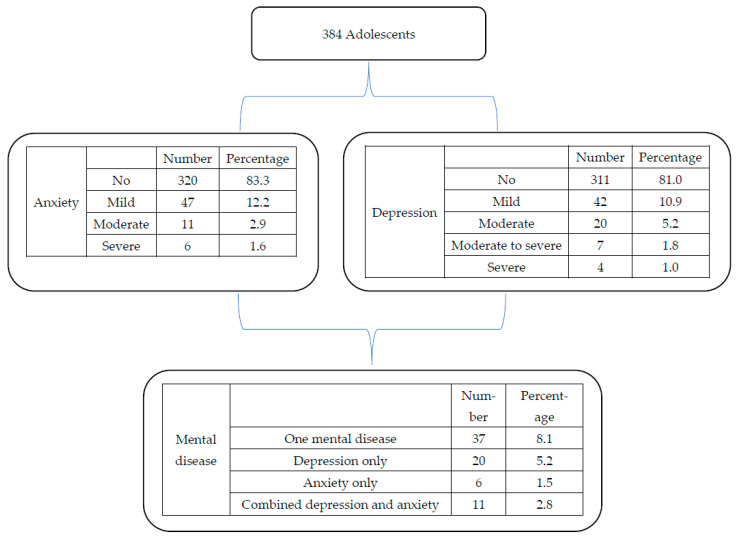
Summary of the results of anxiety and depression among adolescents.

Of the total 384 adolescents, 311 (81.0%), 42 (10.9%), 20 (5.2%), 7 (1.8%), and 4 (1.0%) had no, mild, moderate, moderate to severe, and severe depression, respectively (Table 1). Of the total 384 adolescents, 31 (8.1%) had moderate and or severe depression, depression score ≥ 10 (i.e., necessitate intervention). Of the total depressed participants (number = 31), 11 (35.5%) had anxiety, i.e., co-occurrence of depression and anxiety. Of the 384 adolescents, 37 (9.6%) had at least one mental disorder, i.e., 20 (5.2%) participants with depression only, 6 (1.5%) participants only with anxiety, and 11 (2.8%) participants with comorbidity (both depression-anxiety) (Table 2, Figure 1).

In univariate logistic regression analysis, the mother’s education and occupation, the father’s education and occupation, and the family history of mental disorders, cigarette smoking, and anemia were not associated with depression. Being female, having an older participants’ age, having a higher BMI for age Z-score, and having anxiety were associated with depression (Table 2).

In multivariable logistic regressions, there was no association between age, father’s education, and BMI for age Z-score and depression. However, being female (AOR = 5.35, 95% CI 1.67–17.09) and having anxiety (AOR = 25.98, 95% CI 7.68–87.90) were associated with increased odds of depression (Table 2).

### 3.3. Prevalence and Factors Associated with Anxiety

Of the total 384 adolescents, 320 (83.3%), 47 (12.2%), 11 (2.9%), and 6 (1.6%) had no mild, moderate, and severe anxiety, respectively (Table 1). Of the total 384 adolescents, 17 (4.4%) had moderate and/or severe anxiety, anxiety score ≥ 10 (i.e., necessitated intervention).

In univariate logistic regression analysis, gender, the mother’s education and occupation, the father’s education and occupation, cigarette smoking, and BMI for age Z-score were not associated with anxiety. Older participant’s age, family history of mental disorders, anemia, and depression were associated with anxiety (Table 3).

In multivariable logistic regressions, there was no association between age, gender, father’s occupation, and BMI for age Z-score and anxiety. Positive family history of mental disorder (AOR = 6.31, 95% CI 1.36–29.27), being anemic (AOR = 3.95, 95% CI 1.19–13.12), and depression (AOR = 29.03, 95% CI 7.52–112.05) were associated with increased odds of anxiety (Table 3).

## 4. Discussion

### 4.1. Main Study Findings

The main finding of the present study was that 1 in 10 adolescents in Northern Sudan had at least one mental disorder (depression, anxiety, or depression-anxiety). In the present study, 8.1% and 4.4% of the adolescents had depression and anxiety that necessitated intervention, respectively.

### 4.2. Prevalence of Anxiety and Depression

This prevalence of 1 in 10 adolescents in Northern Sudan had at least one mental disorder is similar to the WHO’s estimation, i.e., globally, 1 in 7 adolescents experiences a mental disorder (3), because, in the current study, only two disorders were studied (depression and anxiety). In the present study, the prevalence (8.1%) of depression is comparable with the results from a previous study in central Sudan [39]. A community study in Khartoum, central Sudan, which included 272 adolescent girls aged 12–19 years old, revealed that 4.2% of them had major depression and 8.7% had partial depressive syndrome [39]. The prevalence rate (8.1%) of depression in the present study is lower than those reported in Jordan, which reported that 25.7% of high school adolescent girls had severe levels of depression [25]. Moreover, the prevalence rate of depression in this study was lower than that reported in Bangladesh, which ranged from 26.5% to 36.6% [9,13]. In India, a cross-sectional study that included 542 adolescents aged 13–18 years old showed that 40% of adolescents had depressive disorders (7.6% major depressive disorders and 32.5% other depressive disorders) [40]. In China, a cross-sectional study that included 1018 adolescents showed that 23.2% of adolescents had depression [14].

### 4.3. Factors Associated with Depression and Anxiety

In the present study, females were 5.35 times at risk of depression. This is consistent with the results of several previous studies [7,13,41]. In Northwest Ethiopia, a school-based cross-sectional study that included 849 adolescents revealed that being female was a risk factor for depression [7]. Furthermore, a recent study conducted by Klaufus et al. in the Netherlands revealed that depression with suicidal ideation was a significant health concern, especially among girls [41]. Several factors might increase female susceptibility to depressive symptoms, such as iron deficiency anemia among girls [16]; biological factors, such as the variation in ovarian hormone levels and decreases in estrogen [42]; exposure to stressful life events; and bullying victimization in preadolescence [43]. The current study did not show an association between age, parent’s education, occupation, smoking, BMI, and depression. Several previous studies showed that age [44], parents employment [34,45], smoking [9], BMI [14,15] and depression. For example, parental education levels might influence adolescents’ mental health by shaping parenting styles, socioeconomic status, and access to resources. Higher education often correlates with better communication and support, fostering adolescent resilience. Conversely, lower educational attainment may limit parental capacity to address mental health issues effectively, impacting adolescents’ emotional well-being. The lack of influence of parents’ education on adolescents’ mental health in our study could be explained by the poor quality of education [21].

This study showed that 4.4% of the adolescents had anxiety. This prevalence (4.4%) is lower than previously reported in central Sudan (54%) (6) and Northwest Ethiopia (66.7%) [7]. The studied region could explain the low prevalence in our study, as the studied region is a rural community. Using different mental health measurement tools. Also, a cutoff GAD-7 of ≥10 for anxiety was used in this study. However, low cutoff scores of ≥6 and ≥7 were used in South Africa (32) and Hong Kong [46], respectively.

In the present study, while a positive family history of mental disorders was positively associated with anxiety, it was not with depression. Consistent with the present result, a survey by Nakie et al. revealed that having a family history of mental disorders is associated with anxiety rather than depression [7]. A family history of mental health issues can predispose adolescents to similar conditions, influencing their emotional regulation and coping strategies. Genetic factors, combined with environmental stressors, may heighten their vulnerability, leading to an increased risk of developing anxiety, depression, and other mental health disorders during critical developmental stages.

In the present study, anemic adolescents are four times at risk of anxiety compared to non-anemic ones. The coexistence of anxiety and anemia increases the severity of mental health disorders [47]. Consistent with the present result, a study by Lee et al. revealed that anemia is a risk factor for psychiatric disorders, including anxiety [47]. Furthermore, iron supplementation can reduce the risk of psychiatric disorders [47]. Treatment of adolescents’ anemia, especially iron deficiency anemia, is not only improving their mental health but also their academic performance, attention, and concentration [48]. The association between anemia and anxiety may be attributed to several reasons [49]. Anxiety may influence healthy eating habits such as selective eating in children, disordered eating behaviors in adolescents, and the increased consumption of added sugars and saturated fats [49], and iron deficiency may influence mental health disorders, including anxiety, via reducing neurotransmitters such as serotonin, dopamine, and noradrenaline [50]. Anemia could decrease physical activity in adolescents; less physical activity increases the possibility of experiencing symptoms of anxiety compared with those who are physically more active [51].

Our results showed a co-occurrence of depressive and anxiety disorders; anxiety increases the likelihood of depression by 26 times, and depression increases the likelihood of anxiety by 29 times. This result supports the existing literature, including Sudan, regarding the comorbidity of depression and anxiety (depression-anxiety) [8,9,10]. However, this high degree of comorbidity in our result does not appear to be symmetrical, i.e., there is a high influence of depression on the occurrence of anxiety (29% vs. 26%). In Bangladesh, Islam et al. reported that adolescents with depression were 10 times at risk of anxiety and vice versa (symmetrical). Recently, a study addressed the negative impact of comorbidity (depression-anxiety) across childhood and adolescence and its adverse outcomes in young adulthood, such as substance misuse [5]. A systematic review and analysis reported that children and adolescents with comorbidity (depression-anxiety) have unique presentations, greater symptom severity, and treatment resistance in comparison with those who have either mental disorder in isolation [52]. Studies addressed the high influence of peer relational problems and stressful life events on contributing to both depression and anxiety [8,53]. Therefore, preventive approaches, such as early identification of adolescents who experienced peer relational problems and stressful life events and early intervention, may decrease comorbidity.

Our results showed no association between age, gender, parent’s education, occupation, and anxiety. However, several previous studies showed an association between adolescent age [11], female gender [54], employment [34,45], anxiety [7], BMI [14,15], and anxiety.

Our results should be cautiously compared with the results of other studies. First, while our study used the WHO’s definition (cutoff age of 10–19 years old), other studies used different ages of their studied groups (early adolescent, late adolescent, or both children and adolescent). Second, while a cutoff of ≥10 is commonly used for both depression and anxiety in our study, different cutoff scores were used by other studies. In South Africa, while the PHQ-9 of cutoff scores of ≥10 is considered with an optimal sensitivity-specificity balance to detect depression among adolescents, for the GAD-7, cutoff scores of ≥6 is considered with an optimal sensitivity-specificity balance to detect anxiety among adolescents [32]. Moreover, in Hong Kong, a cutoff score was set at ≥7 to detect anxiety among adolescents [46]. Third, there are differences in the tools measuring mental health disorders; other tools are used to assess depression, such as the CES-DC (54), and anxiety, such as SCARED—child version [34]. Fourth, there are differences in sociodemographic characteristics such as residency; while our study focused only on rural communities, other studies focused on rural and urban communities. There is a difference in the prevalence of depression and anxiety among adolescents in rural vs. urban, i.e., urban adolescents are at higher risk compared to rural ones, especially in LMICs [10,13]. This may explain the low prevalence of depression (8.1%) and anxiety (4.4%) in our present study compared to other studies [6,7,9,13,14,25,40]. These contradictory data on the prevalence of depression and anxiety and their associated factors, such as age, gender, parental educational level, and occupational status, is a stimulus factor for researchers to study mental health disorders in their countries/regions to address adolescents’ mental health disorders in precise preventive approaches.

### 4.4. Implications of the Study

The present results have implications for improving adolescents’ mental health since one of the identified factors (anemia) is a preventable factor. The present results can act as a foundation for further studies, especially since the current war in Sudan has severe negative impacts on the population’s mental health, including the most vulnerable group of children and adolescents [19]. It is worth mentioning that, in Africa, not only conflicts and wars negatively influence adolescents’ mental health but also emerging diseases such as Ebola, COVID-19 [55], and HIV [56]. The study recommends specific recommendations, including interventional nutritional programs at early ages and establishing or enhancing schools’ health programs, which are crucial to maintaining adolescents’ health in the present and future. In addition, further high-quality research is needed to guide all involved parties, including decision-makers, to address adolescents’ health, especially mental health disorders, taking into account the current study’s limitations. The results of this study and its proposed recommendations will be communicated to the decision-makers and embedded in the existing policies regarding adolescents’ health.

### 4.5. Limitations of the Study

Due to the nature of the study (a cross-sectional study), it is not easy to establish a causality association, i.e., between different variables. Conducting a longitudinal study will give more clarification regarding the association between depression/anxiety and the studied variables among adolescents. The present study was conducted in Northern Sudan, limiting the generalization of its findings to adolescents in Sudan. The potential for bias exists due to the reliance on self-reported data. No data were collected in this study regarding physical activity, dietary patterns, and parent relationships (together, separated, or divorced), which could influence adolescents’ mental health disorders [4,8,57]. Moreover, no information was collected about whether the students took any sedatives or drugs that might affect their emotional state. Such limitations need to be covered in future research.

## 5. Conclusions

The findings indicate that 1 in 10 adolescents in Northern Sudan experiences at least one mental disorder, encompassing depression, anxiety, or a combination of both. Notably, female adolescents are at a higher risk for depression, while factors such as a family history of mental disorders and anemia significantly contribute to anxiety levels. To address these pressing mental health concerns, it is imperative to implement inclusive and holistic programs that incorporate nutritional support, integrate mental health education into school curricula, and introduce community-based interventions aiming to enhance mental well-being among all adolescents, irrespective of gender. Engaging multiple stakeholders, including government agencies, educational institutions, and families, is crucial for these initiatives to be effective and sustainable. By fostering collaboration among these parties during childhood and adolescence, we can create an environment that supports mental health and promotes overall well-being in the community. Such a comprehensive approach will not only address current mental health issues but also contribute to the long-term resilience and health of future generations.

## Figures and Tables

**Table 1:** Characteristics of the studied adolescents who participated in the study in Northern Sudan (*n* = 384), 2022.

Variable		Median	Interquartile Range
Age, years		15.1	14.0–16.3
Body mass index for age Z-score		−0.6	−1.6–0.3
Hemoglobin, g/dL		13.0	12.3–14.1
Depression score (range 0–27)		1.0	1.0–3.0
Anxiety score (range 0–21)		1.0	1.0–3.0
		Frequency	%
Gender	Male	178	46.4
	Female	206	53.6
Mother’s education level	≥secondary	242	63.0
	<secondary	142	37.0
Father’s education level	≥secondary	251	65.4
	<secondary	133	34.6
Mother’s occupation	Employed	34	8.9
	Housewife	350	91.1
Father’s occupation	Skilled worker	160	41.7
	Unskilled worker	224	58.3
Cigarette smoking	No	372	96.9
	Yes	12	3.1
Family history of mental disorders	No	350	91.1
	Yes	34	8.9
Anemia	No	289	75.3
	Yes	95	24.7
Depression severity	No	311	81.0
	Mild	42	10.9
	Moderate	20	5.2
	Moderate to severe	7	1.8
	Severe	4	1.0
Anxiety severity	No	320	83.3
	Mild	47	12.2
	Moderate	11	2.9
	Severe	6	1.6

**Table 2:** Univariate and multivariable logistic regression analysis of factors associated with depression among adolescents in Northern Sudan, 2022.

Variable		Depression		Univariate Analysis		Multivariate Analysis	
		Yes (*n* = 31) PHQ-9 ≥ 10	No (*n* = 353) PHQ-9 < 10	OR (95% CI)	*p* Value	Adjusted OR (95% CI)	*p* Value
Age, years, median (IQR)		15.9 (15.1–16.4)	15.0 (13.9–16.1)	1.45 (1.13–1.85)	0.003	1.28 (0.95–1.72)	0.111
Body mass index for age Z-score, median (IQR)		0.08 (−0.98–0.58)	−0.73 (−1.69–0.32)	1.40 (1.06–1.85)	0.016	1.42 (0.99–2.03)	0.053
		Frequency (%)	Frequency (%)				
Gender	Male	4 (12.9)	174 (49.3)	Reference			
	Female	27 (87.1)	179 (50.7)	6.56 (2.25–19.14)	0.001	5.35 (1.67–17.09)	0.005
Mother’s education level	≥secondary	19 (61.3)	223 (63.2)	Reference			
	<secondary	12 (38.7)	130 (36.8)	1.08 (0.51–2.30)	0.835		
Father’s education level	≥secondary	24 (77.4)	227 (64.3)	Reference			
	<secondary	7 (22.6)	125 (35.7)	0.53 (0.22–0.1.25)	0.147	0.66 (0.24–1.85)	0.433
Mother’s occupation	Employed	3 (9.7)	3 (8.8)	Reference			
	Housewife	28 (90.3)	322 (91.2)	0.90 (0.26–3.13)	0.866	-	-
Father’s occupation	Skilled	11 (35.5)	149 (42.2)	Reference			
	Unskilled	20 (64.5)	204 (57.8)	1.33 (0.62–2.86)	0.468	-	-
Cigarette smoking	No	30 (96.8)	340 (96.9)	Reference			
	Yes	1 (3.2)	11 (3.1)	1.03 (0.13–8.25)	0.978	-	-
Family history of mental illness	No	3 (9.7)	31 (8.8)	Reference			
	Yes	28 (90.3)	322 (91.2)	1.11 (0.32–3.87)	0.866	-	-
Anxiety	No	20 (64.5)	347 (98.3)	Reference			
	Yes	11 (35.5)	6 (1.7)	31.81 (10.67–94.80)	<0.001	25.98 (7.68–87.90)	<0.001
Anemia	No	21 (67.7)	268 (75.9)	Reference			
	Yes	10 (32.3)	85 (24.1)	1.50 (0.68–3.31)	0.314	-	-

Abbreviations: IQR: interquartile range; 95% CI: 95% confidence interval; OR: odds ratio; PHQ-9; Patient Health Questionnaire-9.

**Table 3:** Univariate and multivariable logistic regression analysis of factors associated with anxiety among adolescents in Northern Sudan (*n* = 384), 2022.

Variable		Anxiety		Univariate Analysis		Multivariate Analysis	
		Yes (*n* = 17) GAD-7 ≥ 10	No (*n* = 367) GAD-7 < 10	OR (95% CI)	*p* Value	Adjusted OR (95% CI)	*p* Value
Age, years, median (IQR)		15.8 (15.2–16.7)	15.1 (13.9–16.3)	1.46 (1.05–2.01)	0.023	1.22 (0.79–1.90)	0.372
Body mass index for age Z-score, median (IQR)		−0.01 (−0.98–0.36)	−0.70 (−01.68–0.36)	1.28 (0.90–1.83)	0.174	1.14 (0.71–1.84)	0.579
		Frequency (%)	Frequency (%)				
Gender	Male	4 (23.5)	174 (47.4)	Reference			
	Female	13 (76.5)	193 (52.6)	2.93 (0.94–9.12)	0.064	1.04 (0.25–4.30)	0.961
Mother’s Education level	≥secondary	10 (58.8)	232 (63.2)	Reference			
	<secondary	7 (41.2)	135 (36.8)	1.20 (0.45–3.23)	0.714	-	-
Father’s education level	≥secondary	12 (70.6)	239 (65.1)	Reference			
	<secondary	5 (29.4)	128 (34.9)	0.78 (0.27–2.26)	0.644	-	-
Mother’s occupation	Employed	1 (5.9)	33 (9.0)	Reference			
	House wife	16 (94.1)	334 (91.0)	1.58 (0.20–12.30)	0.662	-	-
Father’s occupation	Skilled	4 (23.5)	156 (42.5)	Reference			
	Unskilled	13 (76.5)	211 (57.5)	2.40 (0.77–7.51)	0.132	2.71 (0.68–10.79)	0.157
Cigarette smoking	No	1 (5.9)	11 (3.0)	Reference			
	Yes	16 (94.1)	354 (97.0)	2.01 (0.24–16.55)	0.516	-	-
Family history of mental illness	No	4 (23.5)	30 (8.2)	Reference			
	Yes	13 (76.5)	337 (91.8)	3.46 (1.06–11.26)	0.040	6.31 (1.36–29.27)	0.019
Depression	No	6 (35.3)	347 (94.6)	Reference			
	Yes	11 (64.7)	20 (5.4)	31.81 (10.67–94.80)	<0.001	29.03 (7.52–112.05)	<0.001
Anemia	No	8 (47.1)	281 (76.6)	Reference			
	Yes	9 (52.9)	86 (23.4)	3.68 (1.38–9.82)	0.009	3.95 (1.19–13.12)	0.025

Abbreviations: IQR: interquartile range; 95% CI: 95% confidence interval; OR: odds ratio; GAD-7: Generalized Anxiety Disorder-7.

## Data Availability

The data of the present study are available from the corresponding author upon reasonable request.
